# Severity of tricuspid regurgitation predicts risk of recurrence of atrial fibrillation after pulmonary vein isolation

**DOI:** 10.1002/ehf2.15197

**Published:** 2025-03-26

**Authors:** Jan Wintrich, Dimitrios Bismpos, Anika Teusch, Valerie Pavlicek, Patrick Fischer, Felix Mahfoud, Michael Böhm, Christian Ukena

**Affiliations:** ^1^ Department of Internal Medicine III, Cardiology, Angiology and Intensive Care Medicine University Hospital, Saarland University Homburg Germany; ^2^ Department of Internal Medicine II, Cardiology and Angiology, Marien Hospital Herne Ruhr University Bochum Bochum Germany; ^3^ Department of Cardiology University Heart Center, University Hospital Basel Basel Switzerland; ^4^ Cardiovascular Research Institute Basel (CRIB), University Heart Center University Hospital Basel Basel Switzerland

**Keywords:** tricuspid regurgitation, atrial fibrillation, mitral regurgitation, right ventricular function, catheter ablation

## Abstract

**Aims:**

Tricuspid regurgitation (TR) results in right atrial remodelling, thus promoting the formation of a substrate for atrial fibrillation (AF). In turn, AF may cause TR by annulus dilatation. We investigated whether the presence of TR affects the efficacy of pulmonary vein isolation (PVI) for AF.

**Methods and results:**

In patients undergoing PVI, we compared the severity of TR before and 6 months after the procedure. Moderate to severe TR was defined as advanced. Moreover, we investigated whether the severity of TR, at baseline and 6 months after PVI, predicted the recurrence of AF. Out of 320 patients, advanced TR at baseline was documented in 13.1%. Six months after PVI, the proportion of patients with advanced TR (13.1% to 7.2%; *P* < 0.001) decreased significantly. Compared with patients with post‐interventional improvement of advanced TR, right atrial (RA) dilatation at baseline was more pronounced in patients without TR improvement (RA area 20.2 ± 4.4 vs. 26.6 ± 8.3 cm). The presence of advanced TR, particularly without improvement during the follow‐up, increased the risk of AF recurrences compared with patients without advanced TR. Even after propensity‐score matching, TR at baseline remained an independent risk predictor regarding recurrent AF [hazard ratio 2.2 (95% confidence interval, 1.1–4.9); *P* = 0.045]. Advanced MR was not associated with increased risk of AF.

**Conclusions:**

In AF patients undergoing PVI, the presence of advanced TR, particularly without improvement 6 months after the procedure, was associated with an increased risk of AF recurrences.

## Introduction

Pulmonary vein isolation (PVI) has emerged as a cornerstone in the management of atrial fibrillation (AF). However, despite recent technological and procedural advances, the success rate of PVI, defined as freedom of AF, remains at approximately 60%–70%.^1^ Particularly in patients with persistent AF, the recurrence of AF after PVI is common.^2^ Atrioventricular valve regurgitation (AVVR) may represent an important risk factor for the onset and persistence of AF. Besides mitral regurgitation (MR), tricuspid regurgitation (TR) also causes atrial remodelling by volume overload of the right atrium, promoting the formation of an AF substrate.^3^ Therefore, the presence of relevant TR could impact the efficacy of PVI. On the other hand, AF leads to annulus dilatation, begetting or deteriorating TR, recently described as atrial secondary TR (A‐STR).^4^, ^5^ This A‐STR phenotype accounts for only 10%–15% of clinically relevant TR. Particularly in heart failure patients with reduced ejection fraction, a ventricular secondary TR (V‐STR) or an overlapping phenotype is more frequently present.^5^ Although recent data indicate an early and aggressive rhythm control approach in A‐STR is associated with a substantial decrease in TR severity, there are no data from adequately powered randomized trials. Moreover, the freedom from AF after PVI in A‐STR has not been sufficiently studied. As a result, the optimal handling of TR in patients with AF is highly debated, and it remains unclear whether treatment of AF or TR needs to be prioritized.^6^ We aimed at investigating the interaction between TR and AF in patients undergoing PVI treatment due to symptomatic AF. Hence, we analysed the change in TR severity within 6 months after PVI therapy. Furthermore, we studied whether severity of TR predicts the risk of AF recurrences after PVI.

## Methods

### Study subjects and design

This explorative, retrospective analysis enrolled patients with symptomatic AF undergoing PVI for the first time at our institution between 2018 and 2021. Only patients with available echocardiographic data at baseline and 6 months after the ablation were included. At baseline, all patients were interviewed regarding symptoms, comorbidities and medical history (particularly regarding onset of AF). Moreover, all patients received a 12‐lead electrocardiogram (ECG) and a transthoracic echocardiography (TTE). To exclude intracardiac thrombus prior to the PVI treatment, all patients underwent a transesophageal echocardiography or contrast‐enhanced computed tomography. After the PVI procedure, patients were scheduled for follow‐up visits at 6 weeks and 6 months. During both visits, patients' symptoms and potential changes in medication were assessed, and a 12‐lead ECG was recorded. Furthermore, after 6 months, a follow‐up TTE was performed in all patients. The patients were followed up until December 2022.

### PVI procedure

The PVI was either performed with cryoablation or radiofrequency (RF) ablation, which was left at the physicians' discretion. Cryoablation was performed with the 28 mm diameter second‐generation cryo‐balloon (Medtronic Inc., Dublin, Ireland) guided by a circular mapping catheter (Achieve Mapping Catheter, Medtronic Inc.). A minimum of one freeze lasting between 180 and 240 s was delivered at the ostium of each pulmonary vein. The PVI was confirmed by entrance and/or exit block. For RF ablation, the ThermoCool catheter (Biosense Webster, Irvine, CA, USA) was used with guidance from a CARTO three‐dimensional (3D) mapping system (Biosense Webster) using a double Lasso technique. The point‐by‐point RF ablation was performed with a power maximum of 30 W, and the endpoint was a bidirectional conduction block between the left atrium and the pulmonary vein. Patients undergoing redo PVI procedures were excluded. Furthermore, in case of history of right atrial flutter, an additional ablation of the cavotricuspid isthmus was performed.

### Echocardiography

All patients included in our study received a TTE at baseline and after 6 months follow‐up. The TTEs and all analysis were performed by two independent, experienced physicians (J. W. and V. P.) who were blinded to the outcome of PVI. The graduation of AVVR was in accordance with recently published guidelines by the European Society of Cardiology (ESC).^7^ Whenever feasible, we calculated quantitative parameters (EROA by PISA, regurgitation volume and regurgitation fraction). Particularly in patients with mild to moderate TR, PISA measurement was not always feasible. In these cases, biplane vena contracta width served as the primary grading parameter. However, all parameters, qualitative, semiquantitative and quantitative, were checked for consistency and taken into account. In case of inconsistent findings, additional 3D echocardiography was performed. We defined AVVR of at least moderate severity as advanced while mild AVVR was defined as non‐advanced. Patients with torrential AVVR were not included.

### Endpoints

This study primarily aimed at investigating the change in TR severity 6 months after the PVI procedure and the influence of TR severity on the probability of AF recurrences after the PVI procedure.

### Detection of atrial arrhythmic recurrences

All patients were followed up for atrial arrhythmia recurrences in our outpatient clinic for a minimum of 6 months after the procedure (mean duration of follow‐up: 1 year). Any documented AF of at least 30 s after the PVI procedure, excluding a 3 months blanking period, was defined as recurrent AF episode. All patients received a 12 lead ECG prior to discharge during the index hospital stay as well as 6 weeks and 6 months after PVI. Twenty‐four‐hour Holter‐ECG was performed 6 months after PVI.

### Statistical analysis

Continuous variables were tested for normal distribution with the Kolmogorov–Smirnov test and by visual inspection of quantile plots. Normally distributed values are reported as mean ± standard deviation (*SD*, while non‐normally distributed values are reported as median and first to third interquartile range (Q1–Q3). Continuous variables between two groups were compared with the Wilcoxon rank‐sum test while the Kruskal–Wallis test was used for comparing continuous variables between more than three groups. Comparisons for categorical variables were performed with the Pearson *χ* ^2^ test while the McNemar test was used on paired categorical variables. To compare categorical variables between more than 2 groups, results were corrected according to Bonferroni. The survival curves of the patients were calculated by the Kaplan–Meier method and compared with the log‐rank test. Stratified Cox proportional‐hazards regression models were used to calculate hazards ratios (HRs) and associated 95% confidence interval (CI). In the multivariable analysis, we considered established AF risk factors as well as all univariate risk factors with a *P* value of <0.1. The characteristics included in the multivariate analysis comprised diabetes, chronic kidney disease, persistent AF, E/e′, LASVi, significant MR, and TR. Independent predictors of outcome were defined by a multivariate value of *P* < 0.05. Furthermore, we performed a secondary analysis that used propensity‐score matching. In the propensity‐score matching analysis, the nearest‐neighbour method was applied to create a matched control sample. All analyses were performed with SPSS statistical software (version 23.0, IBM Inc., Chicago, IL, USA). All authors had full access to the data and take full responsibility for their integrity.

## Results

### Patient characteristics

**Figure 1:**
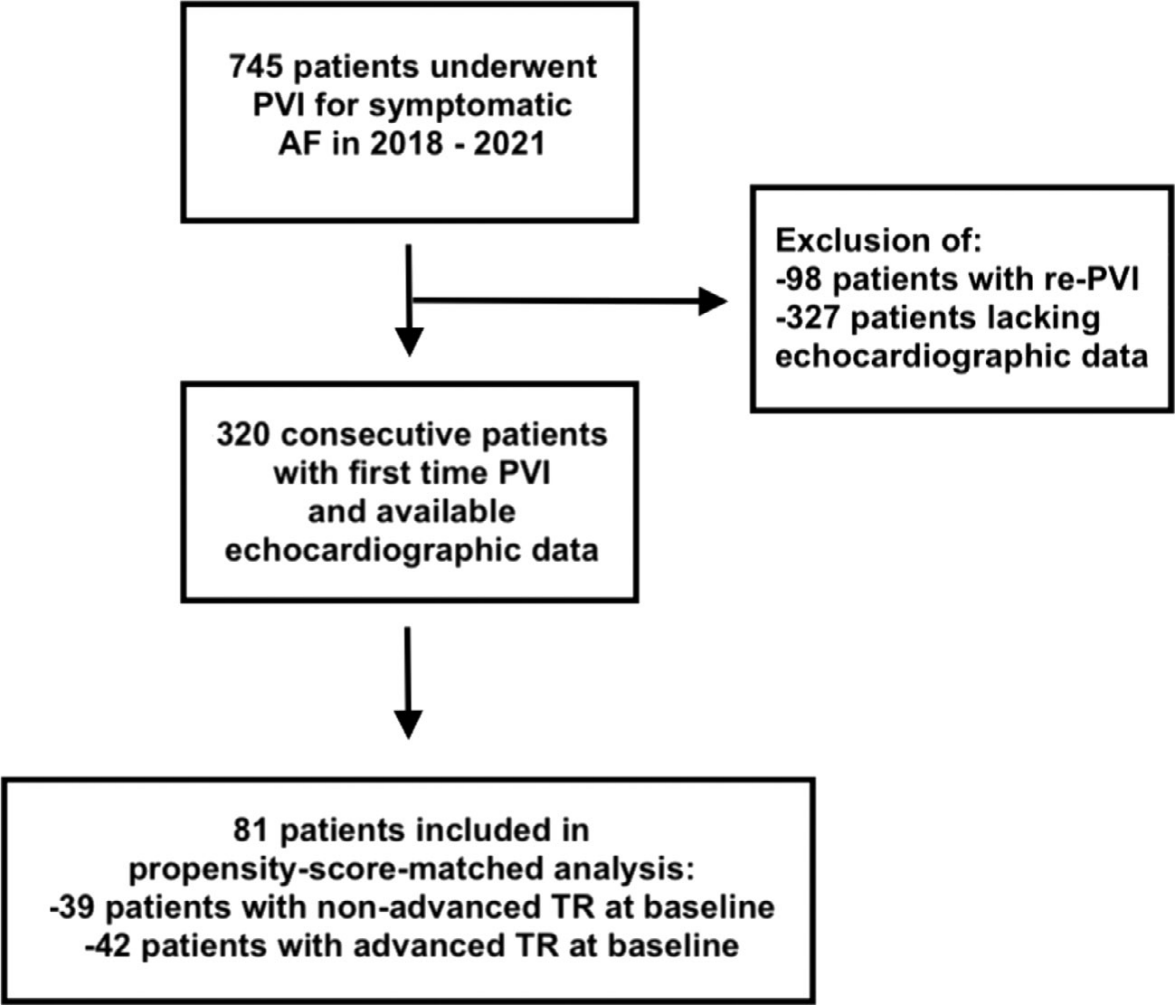
Study design and population.

A total of 320 consecutive patients (mean age 66.3 ± 10 years, 61.6% male, 40% persistent AF) with first time PVI and available echocardiographic data at baseline as well as 6 months after the procedure were included in the study (*Figure* 1). At baseline, most patients had preserved left‐ventricular systolic function (LVEF 55.4 ± 8.3%), dilated atria [right atrial area (RAA) 19.9 ± 5.6 cm^2^ and left atrial systolic volume index (LASVi) 43.6 ± 13.3 mL/m^2^] and normal right‐ventricular longitudinal function (TAPSE 23.5 ± 5 mm) (*Table* 1). Advanced isolated TR was observed in 28 patients (8.7%) while advanced isolated MR was documented in 21 (6.6%) patients (*Table* S1). In 14 patients (4.4%), both advanced MR and TR was present.

**Table 1:** Patient characteristics at the time of the procedure (baseline) in relation to the degree of tricuspid regurgitation.

	All patients (*n* = 320)	Patients without advanced TR (*n* = 278)	Patients with advanced TR (*n* = 42)	*P* (non‐advanced vs. advanced TR)
Age (years)	66.3 ± 10	65.4 ± 10.1	71.9 ± 7.6	<0.001
Male sex (%)	61.6	64.4	42.9	0.007
Persistent AF (%)	40	37.4	57.1	0.015
Diabetes (%)	11.3	9.7	21.4	0.025
CKD (%)	9.1	8.3	21.4	0.207
Obstructive pulmonary disease (%)	7.5	6.5	14.3	0.074
Advanced MR (%)	9.7	7.6	33.3	<0.001
LVEF (%)	55.4 ± 8.3	55.5 ± 8.3	54.5 ± 8.2	0.485
LASVi (mL/m^2^)	43.6 ± 13.3	43.2 ± 13.3	46.4 ± 12.7	0.161
LA reservoir strain (%) (*n* = 113)	18.5 ± 8.3	18.7 ± 8.4	18.4 ± 8.2	0.564
LA conduit strain (%) (*n* = 113)	10 ± 5.3	9.8 ± 5.3	10.1 ± 5.1	0.389
LA contraction strain (%) (*n* = 113)	8.5 ± 3.4	8.7 ± 3.9	8.4 ± 3.3	0.598
E/e′	10.8 ± 5	10.7 ± 4.9	12.0 ± 5.5	0.141
TAPSE (mm)	23.5 ± 5	23.6 ± 5.0	22.3 ± 4.9	0.106
RAA (cm^2^)	19.9 ± 5.6	19.2 ± 4.9	23.5 ± 7.3	<0.001
RV diameter (mm)	39.4 ± 7.6	39.3 ± 7.2	39.7 ± 7.9	0.158
RVSP (mmHg)	27.5 ± 8.8	26.4 ± 7.9	34.2 ± 10.6	<0.001
Cryoablation (%)	66.3	67.6	57.1	0.182
Additional CTI (%)	14.1	12.2	26.2	0.015
Medication at baseline				
ACEi/ARB (%)	63.7	63.3	66.6	0.674
MRA (%)	86.3	86.6	83.3	0.557
BB (%)	19.7	18.3	28.6	0.121
AAD (%)	42.5	42.1	45.2	0.701

*Note*: Normally distributed values are reported as mean ± standard deviation (*SD*), non‐normally distributed values are reported as median and Q1–Q3. Categorical values are reported as *n* (%).

Abbreviations: AAD, antiarrhythmic drug; ACEI, angiotensin‐converting‐enzyme inhibitors; AF, atrial fibrillation; ARB, angiotensin II receptor blocker; BB, beta‐blocker; CKD, chronic kidney disease; CTI, cavotricuspid isthmus; LASVi, left atrial systolic volume index; LVEF, left ventricular ejection fraction; MR, mitral regurgitation; MRA, mineralocorticoid receptor antagonist; RAA, right atrial area; RV, right ventricular; RVSP, right ventricular systolic pressure; TAPSE, tricuspid annular plane systolic excursion TR, tricuspid regurgitation.

Compared with patients with non‐advanced TR, patients with advanced TR were older (71.9 ± 7.6 vs. 65.4 ± 10.1 years; *P* < 0.001), more often female (57.1 vs. 35.6%; *P* = 0.007), and had larger right atria (RAA 23.5 ± 7.3 vs. 19.2 ± 4.9 cm^2^; *P* < 0.001) (*Table* 1). Moreover, diabetes mellitus (21.4 vs. 9.7%; *P* = 0.025), persistent AF (57.1 vs. 37.4%; *P* = 0.015) and a concomitant advanced MR (33.3 vs. 7.6%; *P* < 0.001) were more common in patients with an advanced TR. On the contrary, no significant differences existed between patients with and without advanced TR regarding LVEF (54.5 ± 8.2 vs. 55.5 ± 8.3%; *P* = 0.485) and the left atrium (LASVi 46.4 ± 12.7 vs. 43.2 ± 13.3 mL/m^2^; *P* = 0.161). Furthermore, the percentage of patients treated with a class Ic or III antiarrhythmic agent (45.2 vs. 42.1%; *P* = 0.701) was independent of TR severity. Lastly, the technique of PVI (cryoablation in 57.1 vs. 67.6%; *P* = 0.182) did not differ between patients with and without advanced TR.

A total of three periprocedural complications (one groin hematoma, two temporary injuries of the phrenic nerve) were documented, which did not require further therapy. No adverse events were reported among patients with advanced TR.

### Echocardiographic changes after the PVI

**Figure 2:**
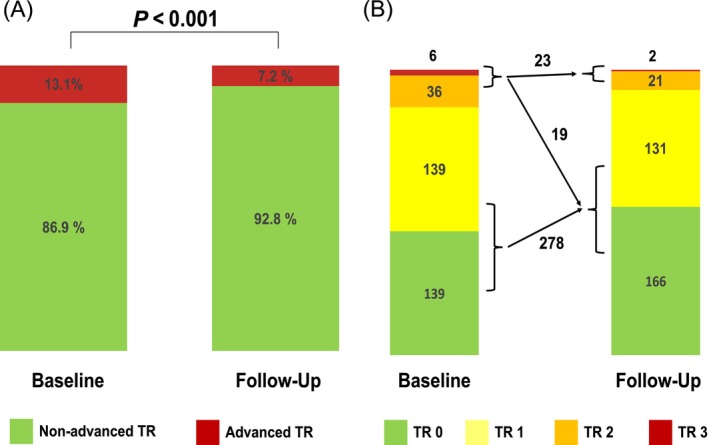
(A,B) Change in the degree of tricuspid regurgitation after pulmonary vein isolation. TR, tricuspid regurgitation.

At 6 months follow‐up, a significant improvement in many echocardiographic parameters, including a decrease in TR severity was detected (Table S2). Out of 42 patients with an advanced TR at baseline, a relevant improvement in TR grade was documented in 19 patients (45%). Hence, the proportion of patients with advanced TR decreased from 13.1% at baseline to 7.2% after PVI treatment (*P* < 0.001) (*Figure* 2). Patients with an improvement of TR severity were more likely to have a smaller right atrium at baseline in comparison with those with persistent significant TR (RAA 20.2 ± 4.4 vs. 26.6 ± 8.3 cm^2^; *P* = 0.002) (*Table* 2). The frequency of concomitant significant MR was comparable between patients with and without an improvement in advanced TR after the PVI procedure (30.4% in patients with persistent advanced TR vs. 36.8% in patients with improved advanced TR, *P* = 0.999).

**Table 2:** Patient characteristics at the time of the procedure (baseline) in relation to the post‐interventional change in the degree of tricuspid regurgitation.

	Patients with stable non‐advanced TR (*n* = 278)	Patients with improved advanced TR (*n* = 19)	*P* vs. stable non‐advanced TR	Patients with persistent advanced TR (*n* = 23)	*P* vs. stable non‐advanced TR	*P* vs. improved TR
Age (years)	65.4 ± 10.1	70.8 ± 8.3	0.058	72.8 ± 7.0	0.002	0.798
Male sex (%)	64.6	36.8	0.049	47.8	0.341	0.999
Persistent AF (%)	37.4	42.1	0.999	69.6	0.007	0.220
Diabetes (%)	9.7	21.1	0.353	21.7	0.216	0.999
CKD (%)	8.3	5.3	0.999	21.7	0.098	0.386
Obstructive pulmonary disease (%)	6.5	15.7	0.376	13.0	0.704	0.999
Advanced MR (%)	7.6	36.8	<0.001	30.4	0.001	0.999
LVEF (%)	55.5 ± 8.3	55.2 ± 8.3	0.985	53.9 ± 8.3	0.684	0.888
LASVi (mL/m^2^)	43.2 ± 13.3	43.7 ± 12.1	0.986	48.8 ± 12.9	0.156	0.454
Mitral E/e′ ratio	10.7 ± 4.9	11.5 ± 3.6	0.820	12.5 ± 6.7	0.291	0.824
TAPSE (mm)	23.6 ± 5	23.2 ± 5.8	0.924	21.5 ± 4.0	0.137	0.551
RAA (cm^2^)	19.2 ± 4.9	20.2 ± 4.4	0.729	26.6 ± 8.3	<0.001	0.002
RVSP (mmHg)	25.4 ± 7.9	36.2 ± 10.7	<0.001	32.7 ± 10.4	0.002	0.364
Cryoablation (%)	67.6%	57.9	0.999	56.5	0.832	0.999
Radiofrequency AF ablation (%)	32.4%	42.1	0.999	43.5	0.999	0.999
Additional CTI ablation (%)	12.2%	10.5	0.999	39.1	0.001	0.108
Sinus rhythm at follow‐up (%)	90.3%	89.5	0.999	69.6	0.008	0.353
Medication at baseline						
ACEi/ARB (%)	63.3%	63.2	0.999	69.6	0.999	0.999
Beta‐blocker (%)	86.7%	73.7	0.346	91.3	0.999	0.382
MRA (%)	18.3%	15.8	0.999	39.1	0.049	0.287
AAD (%)	42.1%	47.3	0.999	43.4	0.999	0.999

*Note*: Normally distributed values are reported as mean ± *SD*, non‐normally distributed values are reported as median and Q1–Q3. Categorical values are reported as *n* (%).

Abbreviations: AAD, antiarrhythmic drug; ACEI, angiotensin‐converting‐enzyme inhibitors; AF, atrial fibrillation; ARB, angiotensin II receptor blocker; CKD, chronic kidney disease; CTI, cavotricuspid isthmus; LASVi, left atrial systolic volume index; LVEF, left ventricular ejection fraction; MR, mitral regurgitation; MRA, mineralocorticoid receptor antagonist; RAA, right atrial area; RVSP, right ventricular systolic pressure; TAPSE, tricuspid annular plane systolic excursion TR, tricuspid regurgitation.

The proportion of advanced MR was also substantially reduced from 10.9% at baseline to 6.6% within 6 months after PVI (*P* < 0.001) (*Figure* S1). Patients with persistent advanced MR were more likely to have a larger left atrium, concomitant TR and diabetes mellitus compared with those with stable MR. However, no parameter predicted the improvement of the MR grade after the ablation (Table S3).

### Recurrence of atrial fibrillation after PVI

**Figure 3:**
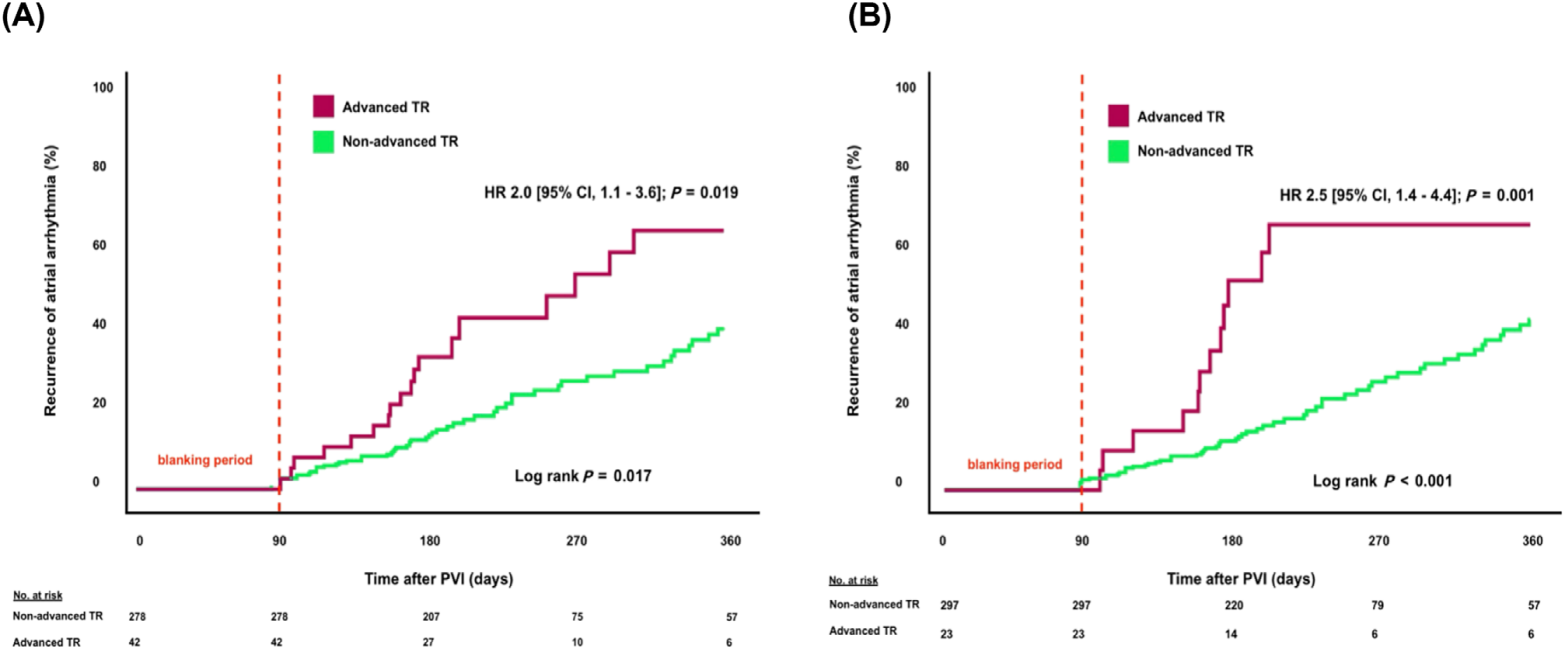
Time to atrial fibrillation recurrence depending on the presence of advanced TR at baseline (A), and at 6 months follow‐up (B). CI, confidence interval; HR, hazard ratio; PVI, pulmonary vein isolation; TR, tricuspid regurgitation.

**Figure 4:**
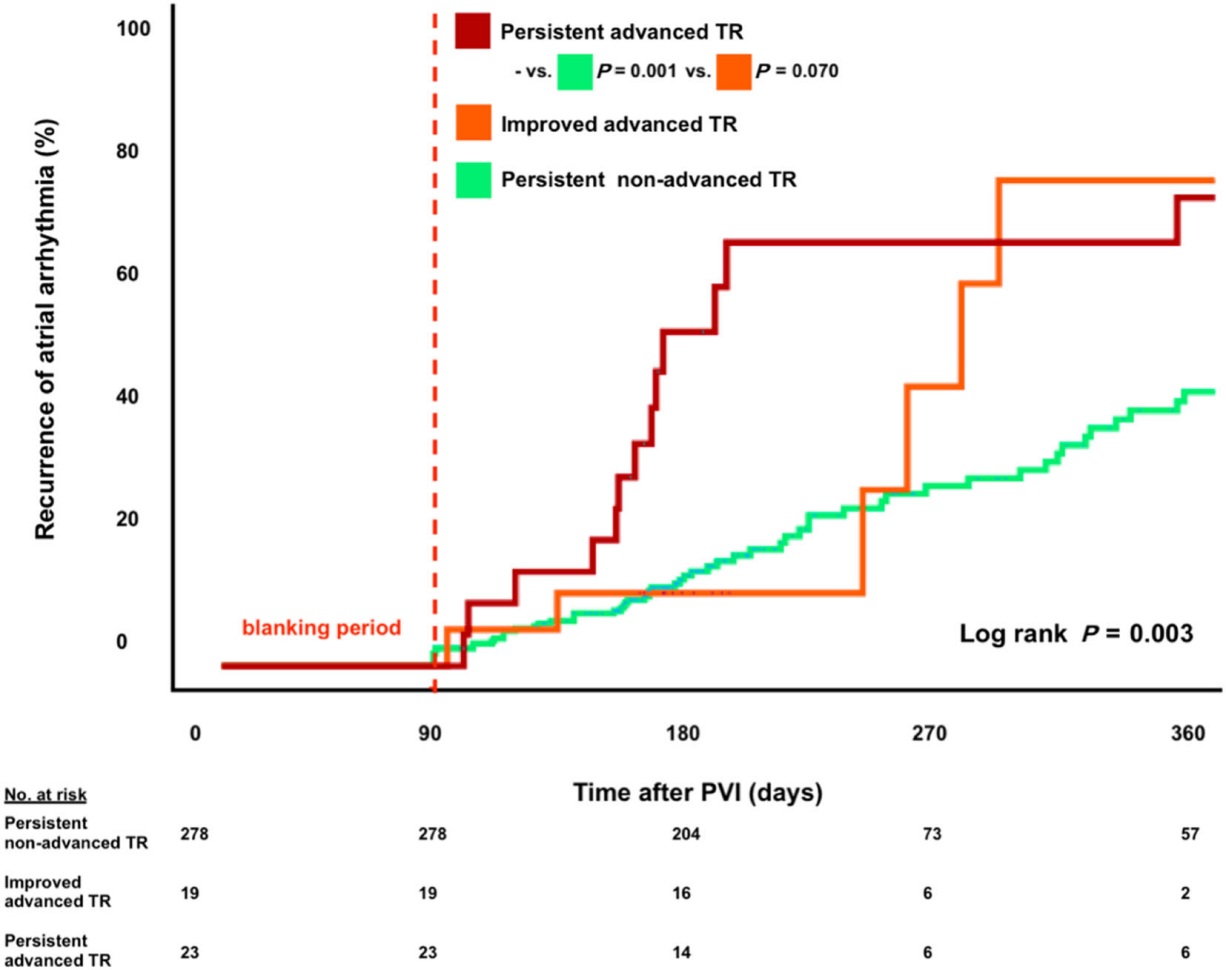
Time to atrial fibrillation recurrence depending on the post‐ablation change in tricuspid regurgitation severity. PVI, pulmonary vein isolation; TR, tricuspid regurgitation.

Recurrent AF after PVI was documented in 104 patients (32.5%). According to a multivariate Cox‐regression analysis (Table S4), the presence of chronic kidney disease [HR 2.32 (95% CI, 1.2–4.5); *P* = 0.012] as well as advanced TR at baseline was associated with an increased risk of AF recurrences after PVI [HR 2.0 (95% CI, 1.1–3.6); *P* = 0.019] (*Figure* 3A). Among patients with advanced TR at baseline, early AF recurrences were particularly often documented in those without a substantial post‐ablation improvement of TR [HR 2.4 (95% CI, 0.9–5.9); *P* = 0.070] (*Figure* 4). Subsequently, compared with baseline, the persistence of advanced TR 6 months after PVI appeared to be an even stronger risk predictor regarding recurrent AF episodes [HR 2.5 (95% CI, 1.4–4.4); *P* = 0.001] (*Figure* 3B).

In contrast, significant MR was neither at baseline [HR 0.7 (95% CI,0.3–1.6); *P* = 0.717] (Figure S2A) nor at follow‐up [HR 1.0 (95% CI,0.5–2.4); *P* = 0.717] (Figure S2B) associated with the recurrence of AF. The post‐interventional change in MR severity did not influence the risk of recurrent arrhythmias (Figure S3).

**Figure 5:**
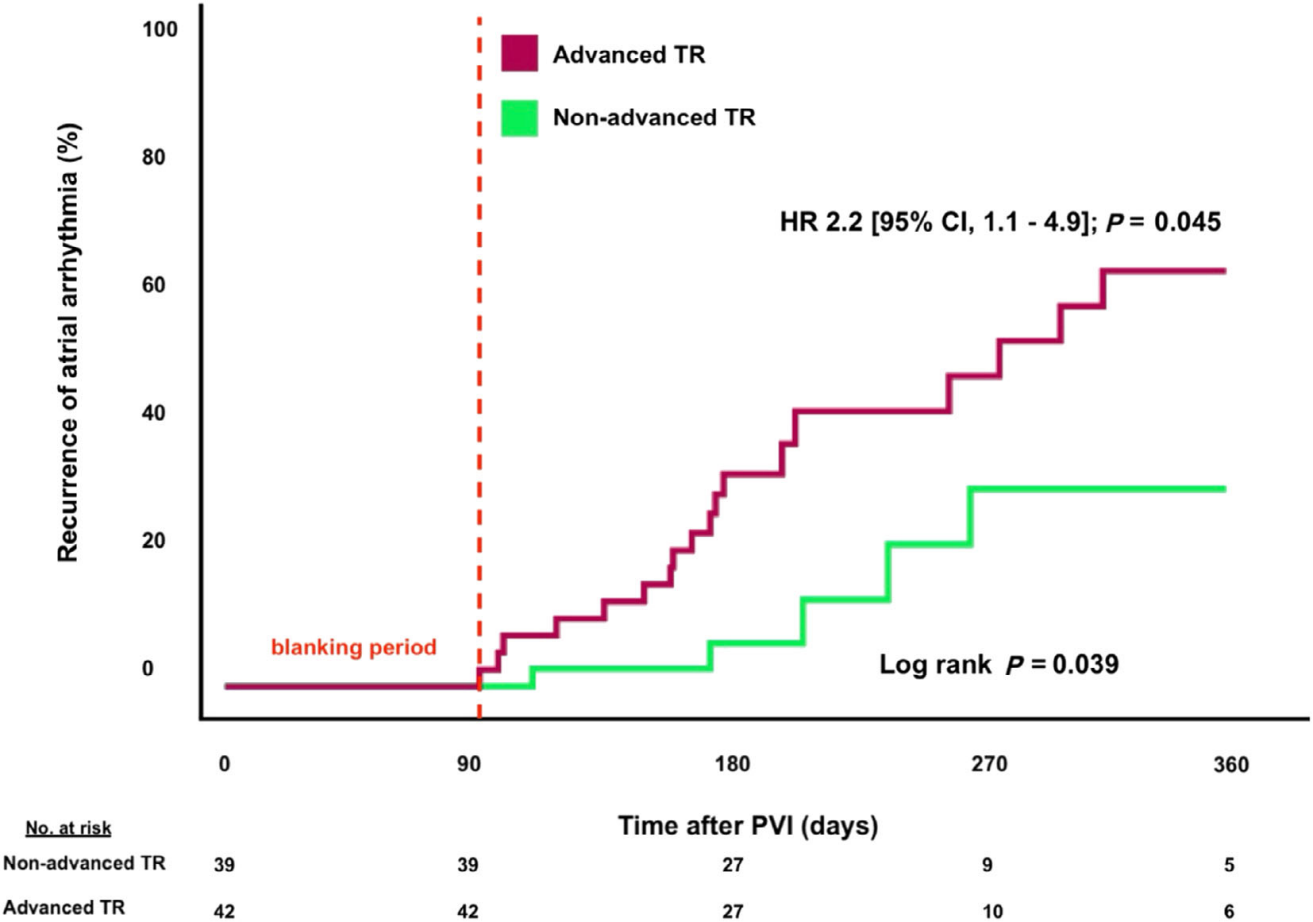
Time to atrial fibrillation recurrence depending on the degree of pre‐interventional tricuspid regurgitation after propensity‐score matching. CI, confidence interval; HR, hazard ratio; PVI, pulmonary vein isolation; TR, tricuspid regurgitation.

To account for potential confounders, we additionally performed a propensity‐score analysis. As shown in *Table* 3, patients with and without advanced TR were matched. After matching, the only difference existed in a higher frequency of diabetes (21.4 vs. 2.6%, *P* = 0.01) and higher RVSP (34.2 ± 10.6 vs. 26.2 ± 8.4 mmHg, *P* < 0.001) in patients with advanced TR. Importantly, concomitant advanced MR was equally distributed in matched patients with and without advanced TR (33.3 vs. 20.5%, *P* = 0.2). Even after propensity score matching, the presence of advanced TR remained an independent risk predictor regarding AF recurrences [HR 2.2 (95% CI, 1.0–4.9); *P* = 0.045] (*Figure* 5).

**Table 3:** Patient characteristics at the time of the procedure (baseline) in relation to the degree of tricuspid regurgitation after propensity‐score matching.

	All patients (*n* = 320)	Patients without advanced TR (*n* = 278)	Patients with advanced TR (*n* = 42)	*P* (non‐advanced vs. advanced TR)	All patients (*n* = 81)	Patients without advanced TR (*n* = 39)	Patients with advanced TR (*n* = 42)	*P* (non‐advanced vs. advanced TR)
Age (years)	66.3 ± 10	65.4 ± 10.1	71.9 ± 7.6	<0.001	70.7 ± 8.5	69.4 ± 9.2	71.9 ± 7.6	0.200
Male sex (%)	61.6	64.4	42.9	0.007	49.4	56.4	42.9	0.228
Persistent AF (%)	40	37.4	57.1	0.015	50.6	43.6	57.1	0.228
Diabetes (%)	11.3	9.7	21.4	0.025	12.3	2.6	21.4	0.010
CKD (%)	9.1	8.3	21.4	0.207	9.9	5.1	14.3	0.172
Obstructive pulmonary disease (%)	7.5	6.5	14.3	0.074	9.9	5.1	14.3	0.172
Advanced MR (%)	9.7	7.6	33.3	<0.001	27.2	20.5	33.3	0.200
LVEF (%)	55.4 ± 8.3	55.5 ± 8.3	54.5 ± 8.2	0.485	53.9 ± 8.9	53.2 ± 9.6	54.5 ± 8.2	0.511
LASVi (mL/m^2^)	43.6 ± 13.3	43.2 ± 13.3	46.4 ± 12.7	0.161	46.9 ± 13.4	47.5 ± 14.3	46.4 ± 12.7	0.705
E/e′	10.8 ± 5	10.7 ± 4.9	12.0 ± 5.5	0.141	11.3 ± 4.7	10.6 ± 3.8	12.0 ± 5.5	0.214
TAPSE (mm)	23.5 ± 5	23.6 ± 5.0	22.3 ± 4.9	0.106	22.7 ± 5.1	23.2 ± 5.3	22.2 ± 4.9	0.436
RAA (cm^2^)	19.9 ± 5.6	19.2 ± 4.9	23.5 ± 7.3	<0.001	22.1 ± 6.7	20.3 ± 5.5	23.5 ± 7.3	0.071
RVSP (mmHg)	27.5 ± 8.8	26.4 ± 7.9	34.2 ± 10.6	<0.001	30.5 ± 10.4	26.2 ± 8.4	34.2 ± 10.6	<0.001
Cryoablation (%)	66.3	67.6	57.1	0.182	65.4	74.4	57.1	0.379
Additional CTI (%)	14.1	12.2	26.2	0.015	22.2	17.9	26.2	0.106
Medication at baseline								
ACEi/ARB (%)	63.7	63.3	66.6	0.674	69.1	71.8	66.7	0.623
MRA (%)	86.3	86.6	83.3	0.557	85.2	87.2	83.3	0.631
BB (%)	19.7	18.3	28.6	0.121	28.4	28.2	28.6	0.971
AAD (%)	42.5	42.1	45.2	0.701	44.4	43.6	45.2	0.883

*Note*: Normally distributed values are reported as mean ± *SD*, non‐normally distributed values are reported as median and Q1–Q3. Categorical values are reported as *n* (%).

Abbreviations: AAD, antiarrhythmic drug; ACEI, angiotensin‐converting‐enzyme inhibitors; AF, atrial fibrillation; ARB, angiotensin II receptor blocker; BB, beta‐blocker; CKD, chronic kidney disease; CTI, cavotricuspid isthmus; LASVi, left atrial systolic volume index; LVEF, left ventricular ejection fraction; MR, mitral regurgitation; MRA, mineralocorticoid receptor antagonist; RAA, right atrial area; RVSP, right ventricular systolic.

Interestingly, in patients with advanced TR at baseline, the presence of atrial flutter at the time of the procedure resulted in a higher risk of AF recurrences [HR 2.7 (95% CI, 1.1–6.9); *P* = 0.039]. This finding was independent of the additional ablation of the cavo‐tricuspid isthmus. Out of all patients, those with advanced TR and documented atrial flutter at baseline had the highest rate of recurrence of atrial arrhythmia (Figure S4). The type of PVI ablation (RF vs. cryo‐balloon), did not influence the post‐ablation outcome in patients with advanced TR at baseline [HR 1.2 (95% CI, 0.38–3.6); *P* = 0.791].

Moreover, in an exploratory analysis, we evaluated the primary endpoint in clinically relevant subgroups, which is highlighted in Figure S5.

## Discussion

In the present study, AF ablation resulted in a relevant improvement of AVVR severity (both TR and MR) in approximately 40% of the patients within 6 months after the procedure. Moreover, the presence of advanced TR at the time of the PVI procedure, and particularly when persistent through 6 months, was associated with an increased risk of AF recurrences. In contrast, advanced MR was not associated with a higher risk of recurrent AF after PVI, neither at baseline nor at 6 months follow‐up,

Secondary TR, which typically develops in the absence of structural abnormalities of the tricuspid valve or its apparatus, accounts for approximately 90% of all cases of TR.^3^, ^8^ The leading mechanism is attributed to tricuspid valve annular dilatation or tricuspid leaflet tethering.^9^ AF belongs to the conditions most often associated with secondary TR.^10^, ^11^ Independent of the presence of left heart disease, AF is associated with right atrial and tricuspid annular remodelling.^12^ Thus, AF promotes right atrial enlargement and dilation of the tricuspid annulus, leading to TR. When examining patients with AF, secondary TR can be found in 12.9% and secondary MR in 7.7% of all patients, respectively.^13^ These incidence rates are in line with our findings.

In AF patients, the presence of persistent AF and its duration, female gender, older age and pulmonary hypertension are associated with the development of significant functional TR.^14^ Furthermore, AF patients with TR are more likely to exhibit biatrial dilatation as well as signs of RV remodelling.^4^ Herein, significant TR at baseline was more likely found in females, older patients with persistent AF and right atrial dilatation.

It has been suggested that the active conversion of AF into sinus rhythm with medical or electrical cardioversion can decrease atrial volumes, annular dimensions, and thus the severity of AVVR.^15^ Previous studies demonstrated beneficial effects on atrial remodelling and subsequently TR severity by catheter ablation of AF. However, there are some important considerations that need to be acknowledged. For instance, one investigation assessed TR severity only by using the vena contracta method and did not report AVVR severity according to the ESC guidelines.^16^ In the meanwhile, two other investigations^6^, ^17^ studied only relatively small cohorts of patients. In our study, a significant number of patients experienced a relevant improvement in AVVR severity. Of note, patients with an improvement in TR severity 6 months after PVI treatment were more likely to be in sinus rhythm than patients without TR improvement. Interestingly, patients with a persistent advanced TR tended to have a more dilated right atrium at baseline. Hence, one could speculate that these patients had already undergone pronounced right atrial remodelling, which could not be affected by AF ablation anymore.

TR may represent an important risk factor for the onset and persistence of AF (Table S6). Future studies need to clarify whether pre‐existence of TR triggers the development of AF or whether long‐lasting AF leads to functional TR by right atrial dilatation. The identification of the leading underlying mechanism can be challenging but may greatly impact individual management strategies.^18^ In an analysis of 239 patients undergoing catheter ablation for AF, the presence of significant functional TR, defined as moderate to severe TR, was an independent predictor for AF recurrences.^13^ In contrast, a similar trial failed to show a significant interaction between 1 year recurrence of AF and TR severity at pre‐ablation echocardiography.^6^ In our analysis, the presence of advanced TR at baseline was associated with a worse post‐interventional outcome in terms of AF recurrences. Moreover, this analysis is the first one to show that the persistence of advanced TR after the PVI appears to be an even stronger predictor for AF recurrence.

Both AF and secondary TR are common comorbidities in patients suffering from heart failure. Just recently, it has been proposed to differentiate between an atrial and ventricular phenotype of secondary TR. According to this new nomenclature, A‐STR is characterized by predominant RA dilatation and preserved biventricular function. In contrast, V‐STR is defined by reduced biventricular function. While A‐STR or an overlapping phenotype is more frequent in heart failure patients with preserved ejection fraction (HFpEF), V‐STR is typically found in heart failure patients with reduced ejection fraction.^5^ In our study, we primarily focused on patients with A‐STR. Hence, the transferability of our results in patients with HFrEF is highly limited.

In the last decades, a plethora of ectopic sources triggering AF were identified outside of the pulmonary veins, for example, vena cavae, crista terminalis and coronary sinus.^19^ Considering the specific features of patients with AF and functional TR, it is tempting to hypothesize that a contrasting, underlying electrophysiological substrate revolving the right atrium may warrant tailored ablation strategies. In our study, the recurrence rate of AF was even higher in patients with a moderate to severe TR and right atrial flutter, even after the additional ablation of the cavo‐tricuspid isthmus. Thus, it could be hypothesized that the presence of atrial flutter is a surrogate for a complex, underlying arrhythmia substrate. As a consequence, further trials to better understand the underlying right atrial substrate as well as to examine possible ablation strategies for patients with moderate to severe TR are needed.

Interestingly, advanced MR was not associated with an increased risk of arrhythmia recurrences, neither at baseline nor at follow‐up. In accordance, Nakamura et al. found only the combination of MR and TR, but not MR itself, to serve as a risk predictor regarding arrhythmia recurrences in 239 AF patients who underwent catheter ablation.^13^ Additionally, in a study by Gertz et al.,^20^ significant MR (defined as ≥moderate) was associated with increased AF recurrences after AF ablation. This is one of the largest studies on the prognostic role of MR in AF patients treated with catheter ablation, which compared 95 patients with and without significant MR, respectively. However, in the multivariate analyses, they only found the left atrial size to be an independent predictor of recurrence, but not MR. In patients with significant MR, the mean left atrial diameter was 45 ± 6.6 mm, and the mean volume index was 34.2 ± 13.0 mL/m^3^. This was significantly higher than in the patient cohort without significant MR, who had a mean left atrial diameter of 41 ± 5 mm and a mean volume index of 27.1 ± 6.7 mL/m^3^. In contrast, in our study, patients without advanced MR comprised a relatively large left atrium with a mean diameter of 44.4 ± 5.9 mm and a mean volume index of 42.7 ± 12.8 mL/m^3^. Hence, one could speculate that MR severity did not predict risk of AF recurrence in our study due to the present left atrial dilatation in both patients with and without advanced MR. Lastly, but most importantly, the number of patients with advanced MR in our study might be too low to reliably assess the prognostic effects of advanced MR on AF recurrences after catheter ablation.

## Limitations

First of all, 6 months might be not enough time to assess beneficial effects of PVI treatment on atrial remodelling and therefore AVVR severity. Furthermore, as already mentioned, the proportion of patients with advanced MR as well as TR was rather low, thus affecting the generalizability of the findings. In addition, we primarily studied patients with A‐STR. Thus, our results may not apply to patients suffering from V‐STR. Furthermore, we did not take into consideration possible medication changes or other clinical entities that could have altered the severity of AVVR. Additionally, the duration of the follow‐up was rather short, and recurrences as well as burden of atrial arrhythmia were not followed closely (e.g., implantable loop recorder), and thus, we could not fully assess the effect of AVVR on atrial arrhythmia recurrence in the long term. Moreover, this is a single‐centre study with a rather small number of patients with significant TR. Thus, our findings can only be regarded as hypothesis generating. Ultimately, larger trials, particularly multi‐centre and randomized trials, are needed to confirm the results. Lastly, left atrial strain data were only available in 113 patients and assessed retrospectively. Most likely because of that reason, left atrial strain values did not serve as independent risk predictors regarding AF recurrences in our analysis, which contrasts with other publications.

## Conclusions

In this study, both TR and MR severity decreased in a significant proportion of patients within 6 months after PVI. In addition, patients with moderate to severe TR, particularly at 6 months follow‐up, experienced an increased risk of atrial arrhythmic recurrences. On the contrary, advanced MR did not serve as an independent risk predictor regarding recurrent AF episodes, neither at baseline nor at follow‐up. Persistent advanced TR was particularly documented in patients with pronounced right atrial dilatation, suggesting advanced right atrial remodelling. Future trials are needed to better understand the underlying atrial substrate, and to improve the therapeutic options for these patients.

## Conflict of interest statement

V. P. has received honoraria from Bayer. M. B. has received lecture honoraria and consulting fees from Amgen, Abbott, AstraZeneca, Bayer, Boehringer Ingelheim, Bristol‐Myers Squibb, Medtronic, Novartis, Servier and Vifor. F. M. is supported by the *Deutsche Gesellschaft für Kardiologie* (DGK), *Deutsche Forschungsgemeinschaft* (SFB TRR219, Project‐ID 322900939) and *Deutsche Herzstiftung*. He has received scientific support from Ablative Solutions, Medtronic and ReCor Medical and speaker honoraria/consulting fees from Ablative Solutions, Amgen, AstraZeneca, Bayer, Boehringer Ingelheim, Inari, Medtronic, Merck, ReCor Medical, Servier and Terumo. J. W. has received lecture honoraria from Bristol Myers Squibb. C. U. has received lecture honoraria from Bayer, Bristol Myers Squibb, Medtronic and ReCor Medical.

## Funding

This work has not been funded.

## Supporting information


**Table S1.** Patient characteristics at the time of the procedure (baseline) in relation to the degree of atrioventricular valve regurgitation.
**Table S2.** Changes in echocardiographic parameters 6 months after pulmonary vein isolation.
**Table S3.** Patient characteristics at the time of the procedure (baseline) in relation to the post‐interventional change in the degree of mitral regurgitation.
**Table S4.** Univariate and multivariate cox‐regression analysis.
**Table S5.** Overview on studies regarding the role of tricuspid regurgitation in AF catheter ablation.
**Figure S1.** Change in the degree of mitral regurgitation after pulmonary vein isolation.
**Figure S2a/b.** Time to atrial fibrillation recurrence in relation to the degree of pre.
**Figure S3.** Time to atrial fibrillation recurrence in relation to the post‐ ablation change in mitral regurgitation severity.
**Figure S4.** Time to atrial fibrillation recurrence in relation to the presence of significant tricuspid regurgitation and atrial flutter at baseline.
**Figure S5.** Risk of AF recurrences in clinically relevant subgroups.
