# Supplementary material for: Severity of tricuspid regurgitation predicts risk of recurrence of atrial fibrillation after pulmonary vein isolation
Source: ESC Heart Fail. 2025 Mar 26;12(3):1905–15. doi: 10.1002/ehf2.15197 (PMC12055437; doi:10.1002/ehf2.15197)
Supplement: Supplementary file 1 — Table S1. Patient characteristics at the time of the procedure (baseline) in relation to the degree of atrioventricular valve regurgitation. Table S2. Changes in echocardiographic parameters 6 months after pulmonary vein isolation. Table S3. Patient characteristics at the time of the procedure (baseline) in relation to the post‐interventional change in the degree of mitral regurgitation. Table S4. Univariate and multivariate cox‐regression analysis. Table S5. Overview on studies regarding the role of tricuspid regurgitation in AF catheter ablation. Figure S1. Change in the degree of mitral regurgitation after pulmonary vein isolation. Figure S2a/b. Time to atrial fibrillation recurrence in relation to the degree of pre. Figure S3. Time to atrial fibrillation recurrence in relation to the post‐ ablation change in mitral regurgitation severity. Figure S4. Time to atrial fibrillation recurrence in relation to the presence of significant tricuspid regurgitation and atrial flutter at baseline. Figure S5. Risk of AF recurrences in clinically relevant subgroups. [file EHF2-12-1905-s001.pdf]

Severity of tricuspid regurgitation predicts risk of recurrence of atrial fibrillation after pulmonary vein isolation

Jan Wintrich[1], Dimitrios Bismpos[1], Annika Teusch[1], Valerie Pavlicek[1], Patrick Fischer[1], Felix Mahfoud[1], Michael Böhm[1], Christian Ukena[1]

Supplemental Material

Supplement Table Legend

Supplement Table 1. Patient characteristics at the time of the procedure (baseline) in relation to the degree of atrioventricular valve regurgitation. Normally-distributed values are reported as mean±standard deviation (SD), non-normally distributed values are reported as median and Q1-Q3. Categorial values are reported as n (%). Abbreviations: AAD=antiarrhythmic drug; ACEI=Angiotensin-converting-enzyme inhibitors; AF=atrial fibrillation; ARB=Angiotensin II receptor blocker; AVVR=atrioventricular valve regurgitation; CKD=chronic kidney disease; CTI= cavotricuspid isthmus; LASVi=left atrial systolic volume index; LVEF=left ventricular ejection fraction; MR=mitral regurgitation; MRA=mineralocorticoid receptor antagonist; RAA=right atrial area; RVSP=right ventricular systolic pressure; TAPSE=tricuspid annular plane systolic excursion TR=tricuspid regurgitation.

Supplement Table 2. Changes in echocardiographic parameters 6 months after pulmonary vein isolation. Normally-distributed values are reported as mean±SD, non-normally distributed values are reported as median and Q1-Q3. Categorial values are reported as n (%). Abbreviations: LAD=left atrial diameter; LASVi=left atrial systolic volume index; LVEF=left ventricular ejection fraction; MR=mitral regurgitation; RAA=right atrial area; RVSP=right ventricular systolic pressure; TAPSE=tricuspid annular plane systolic excursion; TR=tricuspid regurgitation.

Supplement Table 3. Patient characteristics at the time of the procedure (baseline) in relation to the post-interventional change in the degree of mitral regurgitation. Normally-distributed values are reported as mean±SD, non-normally distributed values are reported as median and Q1-Q3. Categorial values are reported as n (%). Abbreviations: AAD=antiarrhythmic drug; ACEI=Angiotensin-converting-enzyme inhibitors; ARB=Angiotensin II receptor blocker; AF=atrial fibrillation; CKD=chronic kidney disease; CTI= cavotricuspid isthmus; LASVi=left atrial systolic volume index; LVEF=left ventricular ejection fraction; MR=mitral regurgitation; MRA=mineralocorticoid receptor antagonist; RAA=right atrial area;

RVSP=right ventricular systolic pressure; TAPSE=tricuspid annular plane systolic excursion TR=tricuspid regurgitation.

Supplement Table 4. Univariate and multivariate cox-regression analysis. Normally-distributed values are reported as mean±SD, non-normally distributed values are reported as median and Q1-Q3. Categorial values are reported as n (%). Abbreviations: AAD=antiarrhythmic drug; ACEI=Angiotensin-converting-enzyme inhibitors; ARB=Angiotensin II receptor blocker; AF=atrial fibrillation; CKD=chronic kidney disease; CTI= cavotricuspid isthmus; LASVi=left atrial systolic volume index; LVEF=left ventricular ejection fraction; MR=mitral regurgitation; MRA=mineralocorticoid receptor antagonist; RAA=right atrial area; RVSP=right ventricular systolic pressure; TAPSE=tricuspid annular plane systolic excursion TR=tricuspid regurgitation.

Supplement Table 5. Overview on studies regarding the role of tricuspid regurgitation in AF catheter ablation. Abbreviations: AAD=antiarrhythmic drug; AT=atrial tachycardia; AF=atrial fibrillation; LA=left atrium; LASVi=left atrial systolic volume index; MR=mitral regurgitation; RA=right atrium; TTE=transthoracic echocardiography; TR=tricuspid regurgitation.

Supplement Table 1. Patient characteristics at the time of the procedure (baseline) in relation to the degree of atrioventricular valve regurgitation

Characteristics	Patients without advanced AVVR (n=257)	Patients with advanced TR only (n=28)	p (Non-advanced vs. advanced TR)	Patients with advanced MR only (n=21)	p (Non-advanced vs. advanced MR)	Patients with advanced MR and TR (n=14)	p (Non-advanced vs. advanced MR and TR)
Age (years)	65.4 ± 10.1	71.3 ± 8.1	**0.016**	65.4 ± 10.5	0.999	73.0 ± 6.6	**0.028**
Male sex (%)	64.2	50	0.839	66.6	0.999	28.5	**0.044**
Persistent AF (%)	37.7	53.5	0.621	33.3	0.999	64.2	0.285
Arterial hypertension (%)	78.9	82.1	0.999	85.7	0.999	71.4	0.999
Diabetes (%)	8.6	17.8	0.664	23.8	0.140	28.6	0.08
CKD (%)	8.6	17.8	0.664	4.7	0.999	7.1	0.999
Obstructive pulmonary disease (%)	7	14.3	0.511	0	n/a	14.3	0.931
LVEF(%)	55.7 ± 8.3	54.1 ± 8.5	0.783	53.4 ± 8.0	0.638	55.4 ± 7.9	0.999
LASVi (ml/m²)	42.5 ± 12.8	44.8 ± 6.9	0.828	52.6 ± 15.5	**0.005**	49.4 ± 14.1	0.220
Mitral e/e' ratio	10.6 ± 5.0	12.6 ± 6.6	0.311	11.4 ± 3.9	0.910	11.1 ± 3.1	0.984
TAPSE (mm)	23.7 ± 5.1	22.3 ± 5.4	0.527	23.0 ± 3.8	0.933	22.2 ± 3.9	0.711
RAA (cm²)	19.1 ± 4.9	23.9 ± 7.2	**0.001**	19.9 ± 5.6	0.945	22.9 ± 7.8	0.074
RVSP (mmHg)	26.4 ± 8.1	32.4 ± 9.8	**0.002**	25.7 ± 6.2	0.983	37.9 ± 11.2	**<0.001**
Cryoablation (%)	67.3	71.4	0.999	60.7	0.999	50	0.999
Radiofrequency AF ablation (%)	32.7	28.6	0.999	39.3	0.999	50	0.999
Additional CTI ablation (%)	11.3	23.8	0.228	25	0.553	28.6	0.324
Medication at baseline							
ACEi/ARB (%)	63	67.9	0.999	66.6	0.999	64.3	0.999
Beta blocker (%)	87.2	80.9	0.999	78.6	0.999	92.8	0.999
MRA (%)	17.9	23.8	0.999	35.7	0.146	14.3	0.999
AAD (%)	41.6	47.6	0.999	53.6	0.999	28.6	0.999

Supplement Table 2. Changes in echocardiographic parameters 6 months after pulmonary vein isolation

	Baseline	Follow-Up	p
Advanced TR	42 (13.1%)	23 (7.2%)	**<0.001**
Advanced MR	35 (10.9%)	21 (6.6)	**<0.001**
LAD (mm)	44.5 ± 5.9	43.8 ± 7.1	**0.011**
LASVi (ml/m²)	43.6 ± 13.2	39.9 ± 12.1	**<0.001**
LVEF(%)	55.3 ± 8.2	57 ± 6.9	**<0.001**
Mitral e/e' ratio	10.8 ± 5	10.8 ± 5.1	0.845
TAPSE (mm)	23.5 ± 5	23.6 ± 4.5	0.49
RAA (cm²)	19.8 ± 5.6	18.7 ± 5.6	**<0.001**
RVSP (mmHg)	27.5 ± 8.7	26.1 ± 7.9	**0.01**

Supplement Table 3. Patient characteristics at the time of the procedure (baseline) in relation to the post-interventional change in the degree of mitral regurgitation

	Patients with stable non-advanced MR (n=278)	Patients with improved advanced MR (n=19)	p vs. stable n.ad. MR	Patients with persistent advanced MR (n=23)	p vs. stable n.ad. MR	p vs. improved MR
**Age (years)**	65.4 ± 10.1	70.8 ± 8.3	0.058	72.8 ± 7.0	**0.002**	0.798
**Male sex (%)**	64.6	36.8	**0.049**	47.8	0.341	0.999
**Persistent AF (%)**	37.4	42.1	0.999	69.6	**0.007**	0.220
**Diabetes (%)**	9.7	21.1	0.353	21.7	0.216	0.999
**CKD (%)**	8.3	5.3	0.999	21.7	0.098	0.386
**Obstructive pulmonary disease (%)**	6.5	15.7	0.376	13.0	0.704	0.999
**Advanced TR (%)**	7.6	36.8	**<0.001**	30.4	**0.001**	0.999
**LVEF(%)**	55.5 ± 8.3	55.2 ± 8.3	0.985	53.9 ± 8.3	0.684	0.888
**LASVi (ml/m²)**	43.2 ± 13.3	43.7 ± 12.1	0.986	48.8 ± 12.9	0.156	0.454
**Mitral e/e' ratio**	10.7 ± 4.9	11.5 ± 3.6	0.820	12.5 ± 6.7	0.291	0.824
**TAPSE (mm)**	23.6 ± 5	23.2 ± 5.8	0.924	21.5 ± 4.0	0.137	0.551
**RAA (cm²)**	19.2 ± 4.9	20.2 ± 4.4	0.729	26.6 ± 8.3	**<0.001**	**0.002**
**RVSP (mmHg)**	25.4 ± 7.9	36.2 ± 10.7	**<0.001**	32.7 ± 10.4	**0.002**	0.364
**Cryoablation (%)**	67.6%	57.9	0.999	56.5	0.832	0.999
**Radiofrequency AF ablation (%)**	32.4%	42.1	0.999	43.5	0.999	0.999
**Additional CTI ablation (%)**	12.2%	10.5	0.999	39.1	**0.001**	0.108
**Sinusrhythm at follow-up (%)**	90.3%	89.5	0.999	69.6	**0.008**	0.353
**Medication at baseline**						
**ACEi/ARB (%)**	63.3%	63.2	0.999	69.6	0.999	0.999
**Beta blocker (%)**	86.7%	73.7	0.346	91.3	0.999	0.382
**MRA (%)**	18.3%	15.8	0.999	39.1	0.049	0.287
**AAD (%)**	42.1%	47.3	0.999	43.4	0.999	0.999

Supplement Table 4. Univariate and multivariate cox-regression analysis

Univariate Analysis	p	HR, 95% CI	Multivariate Analysis	p	HR, 95% CI
Age	0.713	0,92 (0.62-1.38)			
Persistent AF	0.077	1.42 (0.96-2.09)	Persistent AF	0.453	1.19 (0.75-1.9)
Chronic kidney disease	0.131	1.4 (0.88-2.69)	Chronic kidney disease	**0.012**	**2.32 (1.2-4.48)**
Diabetes Mellitus	0.891	1.04 (0.9-1.83)	Diabetes Mellitus	0.872	1.0 (0.6-1.95)
Arterial hypertension	0.944	0.98 (0.6-1.59)			
LA enlargement	0.352	1.21 (0.81-1.80)	LA enlargement	0.285	1.3 (0.8-2.11)
Diastolic dysfunction	0.123	1.49 (0.89-2.5)	Diastolic dysfunction	0.759	1.09 (0.61-1.94)
RA enlargement	0.609	1.22 (0.56-2.7)			
Pulmonary hypertension by RVSP	0.52	1.24 (0.64-2.4)			
Advanced MR	0.807	0.7 (0.36-1.62)	Advanced MR	0.403	0.71 (0.32-1.56)
Advanced TR	0.017	2 (1.1-3.6)	Advanced TR	**0.019**	**2 (1.11-3.59)**
Advanced MR and TR	0.529	1.38 (0.55-3.77)			

Supplement Table 5. Overview on studies regarding the role of tricuspid regurgitation in AF catheter ablation

Publication	Aim	Design	Type of AF	No. of patients	Follow-up	Results
**Nishiwaki, 2023 (6)**	Evaluation of the effectiveness of catheter ablation as well as the mechanisms of improvement of atrial functional TR	Retrospective, single center	Paroxysmal or persistent, only patients with moderate or greater TR	102	12 months	No significant difference between one-year recurrence of AF/AT and TR severity at pre-ablation TTE
**Nakamura, 2021 (13)**	Evaluation of the impact of functional MR and TR in AF recurrence after catheter ablation	Prospective, single center	Paroxysmal or non-paroxysmal	194	12 months	Significant functional TR was an independent predictor of arrhythmia recurrence after ablation, especially in the concomitant presence of functional MR. Functional MR alone yielded no significant differences
**Markman, 2020 (17)**	Determination of the effect of restoring sinus rhythm through catheter ablation of AF on the degree of TR	Retrospective, single center	Paroxysmal or persistent, only patients with at least moderate TR	36	12 months	Freedom from AF post ablation was associated with a greater likelihood of improvement in TR by at least one grade
**Zhao, 2014 (21)**	Assessment of the role of valvular regurgitation in outcomes of patients receiving catheter ablation	Retrospective, single center	Long standing-persistent, refractory to ≥ 2 AADs	216	19 months	MR, LA size and AF duration were independent predictors of recurrent atrial tachycardias (ATs). Patients with recurrent ATs had greater RA enlargement and TR
**Gunturiz-Beltran, 2022 (22)**	Assessment of the role of RA structural remodeling in the arrhythmogenic substrate of AF using cardiac magnetic resonance	Prospective, multi-center	Paroxysmal or persistent	109	24 months	Right atrial sphericity induced by significant TR was a predictor of AF recurrence
**Ukita, 2023 (23)**	Investigation of predictors and outcomes of improvement of TR after radiofrequency catheter ablation	Prospective, single center	Persistent	141	12 months	The absence of late-recurrence of AF was independently associated with an improvement of TR after the catheter ablation

Supplement Figure Legends

Supplement Figure 1. Change in the degree of mitral regurgitation after pulmonary vein isolation. Abbreviations: AF=atrial fibrillation; MR=mitral regurgitation; PVI=pulmonary vein isolation.

Supplement Figure 2a/b. Time to atrial fibrillation recurrence in relation to the degree of pre- (a) and post-interventional mitral regurgitation (b). Abbreviations: AF=atrial fibrillation; CI=confidence interval; HR=hazard ratio;MR=mitral regurgitation; PVI=pulmonary vein isolation.

Supplement Figure. Time to atrial fibrillation recurrence in relation to the post- ablation change in mitral regurgitation severity. Abbreviations: Abbreviations: AF=atrial fibrillation; MR=mitral regurgitation; PVI=pulmonary vein isolation.

Supplement Figure 4. Time to atrial fibrillation recurrence in relation to the presence of significant tricuspid regurgitation and atrial flutter at baseline. Abbreviations: AF=atrial fibrillation; PVI=pulmonary vein isolation; TR=tricuspid regurgitation.

Supplement Figure 5. Risk of AF recurrences in clinically relevant subgroups. Moderate/ severe left atrial enlargement is defined as LASVi ≥ 40 ml/m^3. Moderate/ severe RA enlargement is defined as RAA ≥ 22 cm^2. Abbreviations: AF=atrial fibrillation; LA=left atrium; RA=right atrium.

Supplement Figure 1. Change in the degree of mitral regurgitation after pulmonary vein isolation

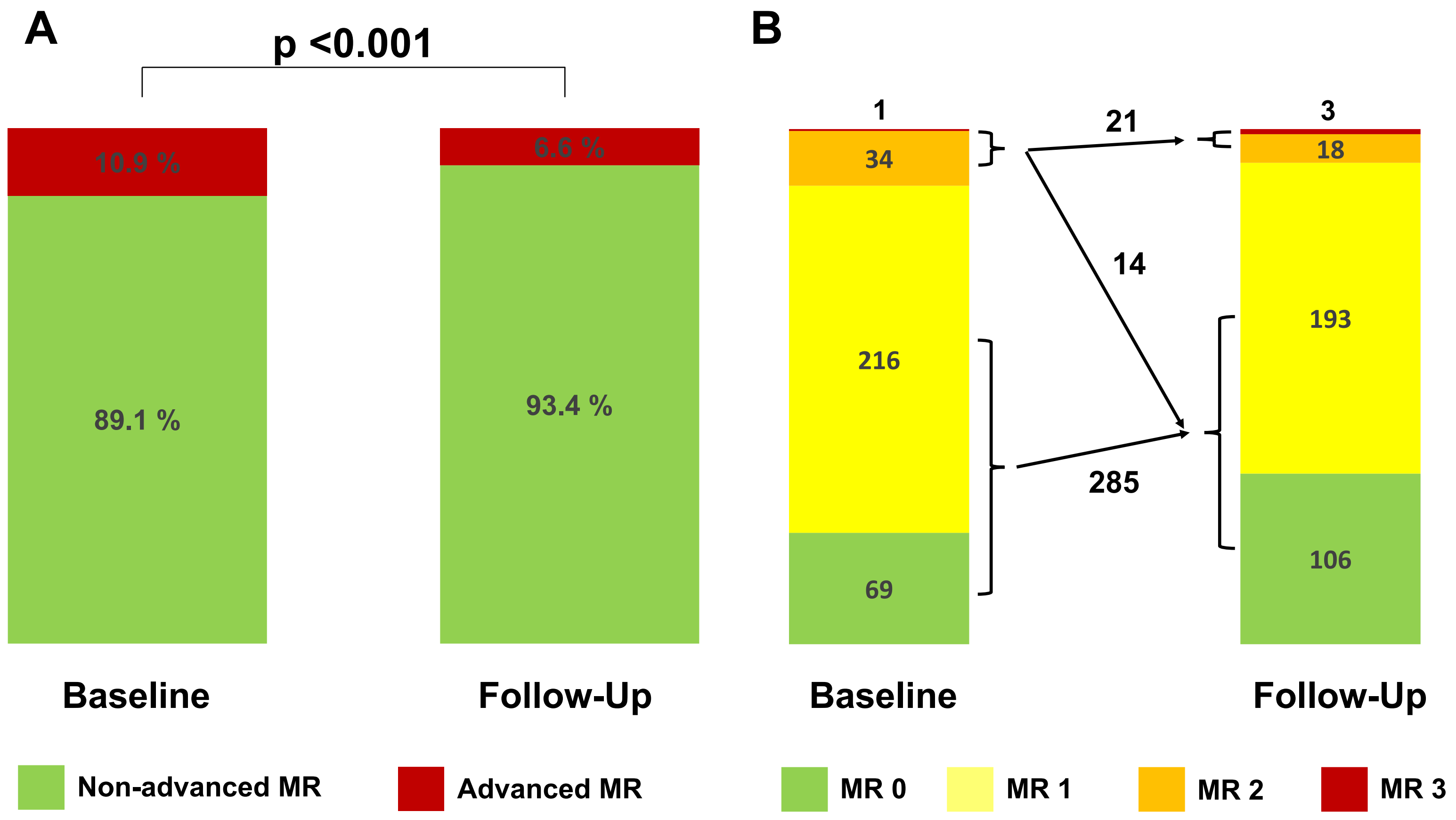

Supplement Figure 2a and 2b. Time to atrial fibrillation recurrence in relation to the degree of pre- (a) and post-interventional mitral regurgitation (b)

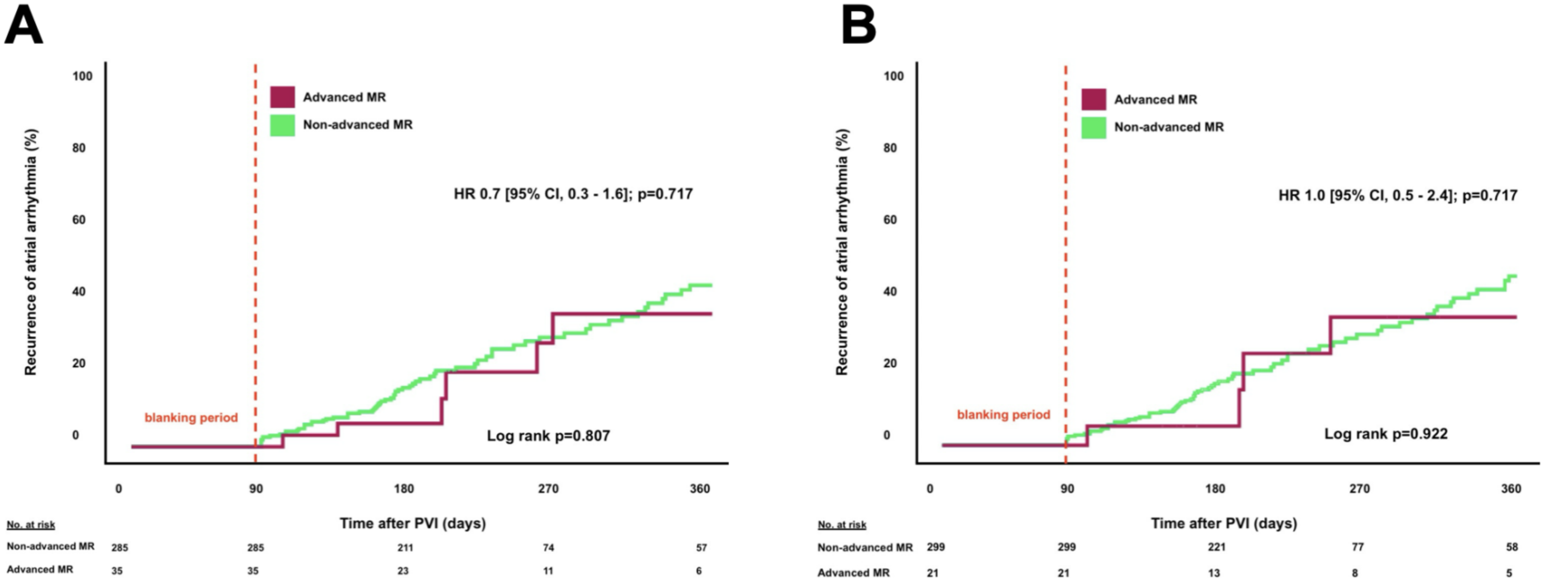

Supplement Figure 3. Time to atrial fibrillation recurrence in relation to the post-ablation change in mitral regurgitation severity

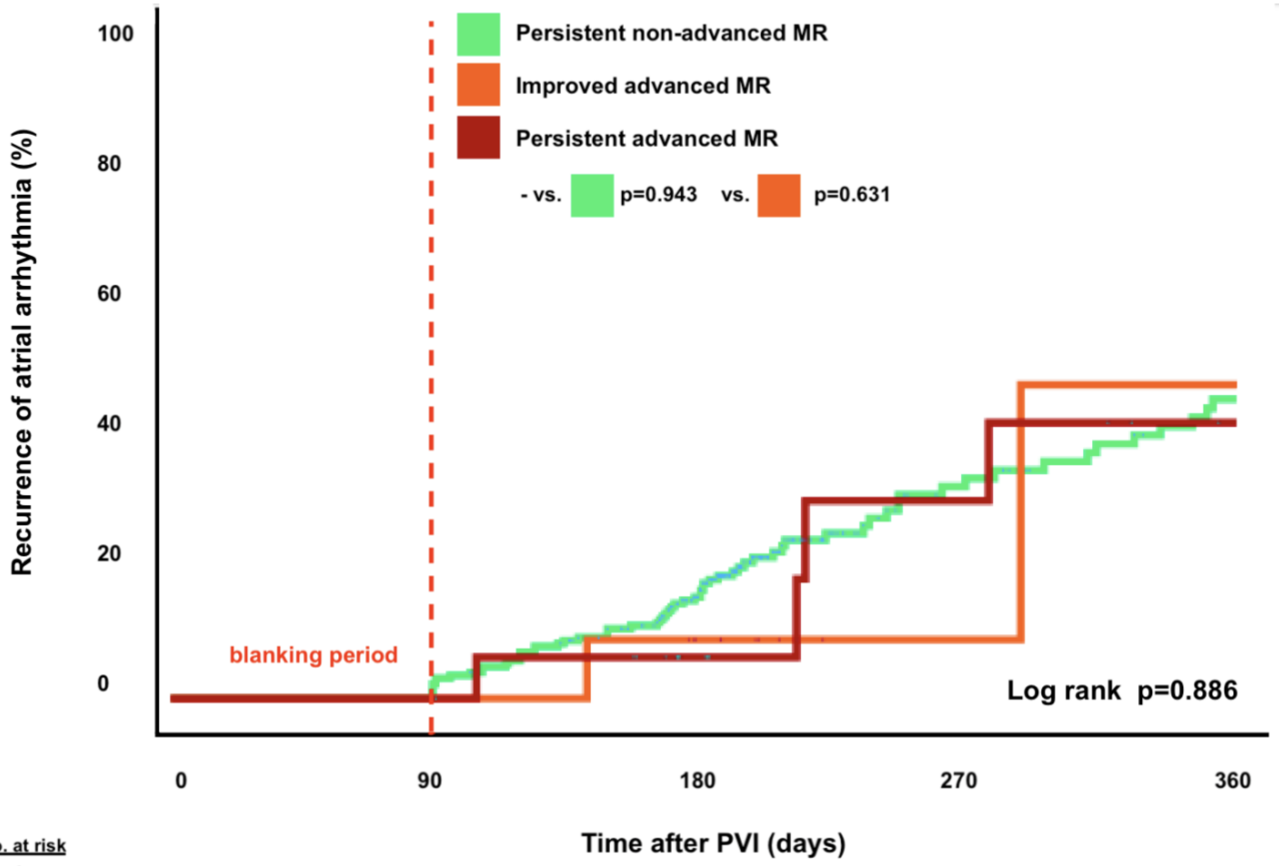

No. at risk	0	90	180	270	360
Persistent non-advanced MR	285	285	209	74	58
Improved advanced MR	14	14	12	3	2
Persistent advanced MR	21	21	13	8	5

Supplement Figure 4. Time to atrial fibrillation recurrence in relation to the presence of significant tricuspid regurgitation and atrial flutter at baseline

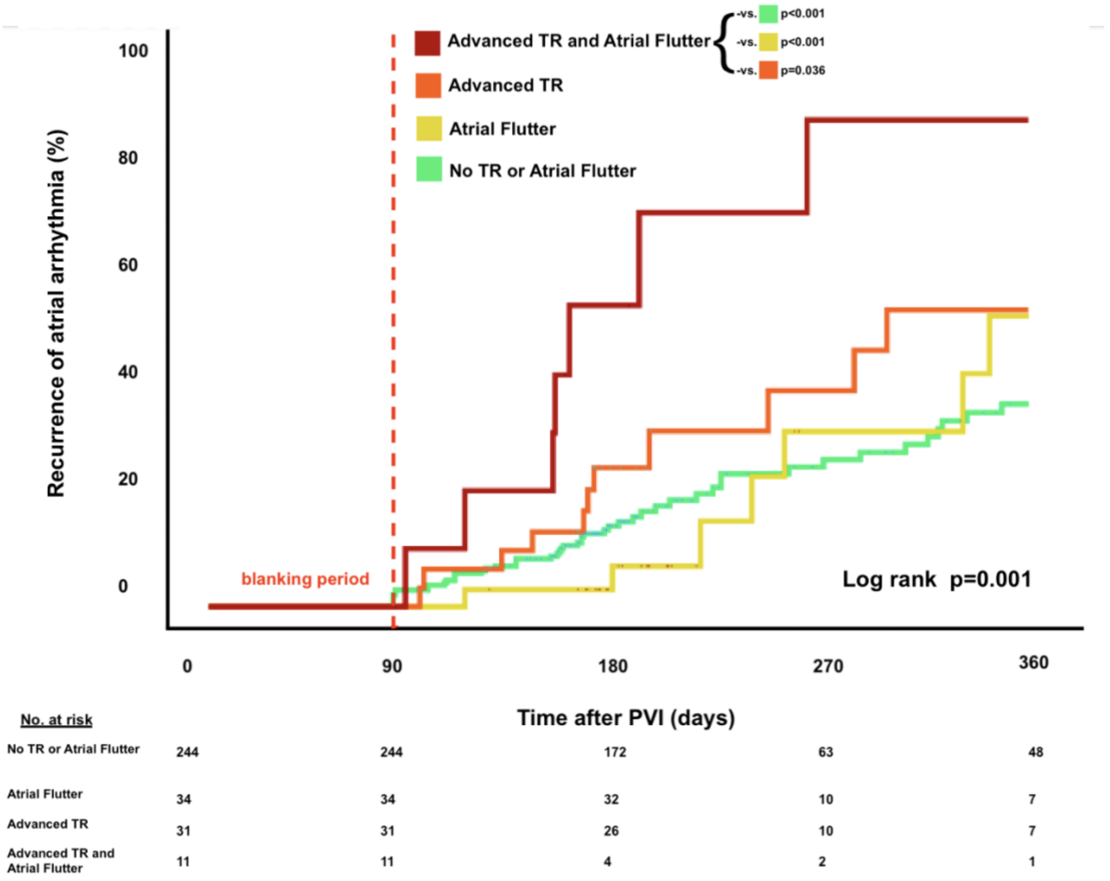

Supplement Table 5. Risk of AF recurrences in clinically relevant subgroups

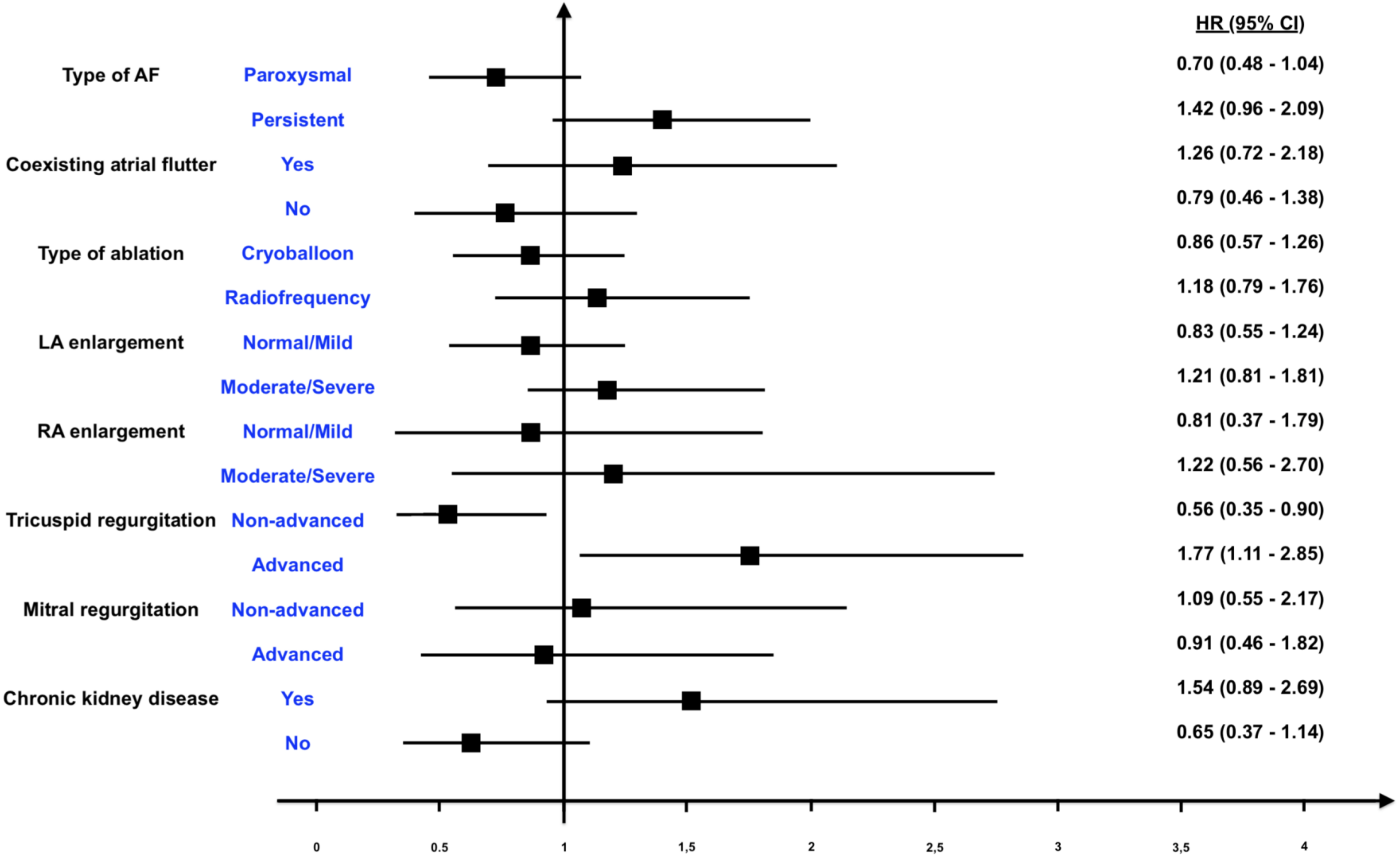

References

21. Zhao L, Jiang W, Zhou L, Gu J, Wang Y, Liu Y, Zhang X, Wu S, Liu X. The role of valvular regurgitation in catheter ablation outcomes of patients with long-standing persistent atrial fibrillation. Europace. 2014 Jun;16(6):848-54.

22. Gunturiz-Beltrán C, Nuñez-Garcia M, Althoff TF, Borràs R, Figueras I Ventura RM, Garre P, Caixal G, Prat-González S, Perea RJ, Benito EM, Tolosana JM, Arbelo E, Roca-Luque I, Brugada J, Sitges M, Mont L, Guasch E. Progressive and Simultaneous Right and Left Atrial Remodeling Uncovered by a Comprehensive Magnetic Resonance Assessment in Atrial Fibrillation. J Am Heart Assoc. 2022 Oct 18;11(20):e026028.

23. Ukita K, Egami Y, Nohara H, Kawanami S, Sugae H, Kawamura A, Nakamura H, Yasumoto K, Tsuda M, Okamoto N, Matsunaga-Lee Y, Yano M, Nishino M, Tanouchi J. Predictors and outcomes of tricuspid regurgitation improvement after radiofrequency catheter ablation for persistent atrial fibrillation. J Cardiovasc Electrophysiol. 2023 Jun;34(6):1360-1366.
